# Asynchronous Bilateral Adnexal Torsion: A Case Report

**DOI:** 10.7759/cureus.39370

**Published:** 2023-05-23

**Authors:** Anjala Nizam, Huda Ahmed, Litty Paulose, Haroutyoun Margossian

**Affiliations:** 1 Department of Medicine and Surgery, Dubai Academic Health Corporation, Dubai, ARE; 2 Department of Obstetrics and Gynecology, Latifa Women and Children Hospital, Dubai, ARE

**Keywords:** asynchronous bilateral ovarian torsion, ovarian torsion, bilateral adnexal torsion, ovarian cystectomy, adnexal torsion

## Abstract

Adnexal torsion is a gynecological emergency that requires immediate surgical intervention to prevent permanent loss of the affected ovary. Here, we present a case of a 25-year-old female who presented to the emergency with a six-day history of lower abdominal pain. A computed tomography scan showed a clinical picture of ovarian torsion, hence, the patient underwent laparoscopic ovarian tissue-sparing right ovarian cystectomy with shortening of the right utero-ovarian ligament for a twisted right ovarian cyst. Intraoperatively, a left pelvic mass was seen sitting freely in the cul-de-sac, which was thought to be the left adnexa that possibly underwent complete torsion in the past, went unnoticed, and got amputated and separated from its pedicle. Postoperatively, the patient had a smooth recovery and was discharged within four days, in stable condition. Three months later, the patient began having her periods, though irregularly.

## Introduction

Adnexal torsion is a serious gynecological emergency where there is a partial or complete rotation of the adnexa around its ligamentous support. This structure contains vascular and lymphatic vessels; hence, upon torsion, the reduction in venous reflux and arterial flow may cause ischemia of the adnexa and ultimately necrosis, which would result in the loss of the afflicted ovary permanently [1]. Adnexal torsion is the fifth most frequent cause of acute pelvic pain in women of reproductive age [2]. It is said to happen at any age, from pre-pubescence to post-menopause, with women in their 20s and 30s having the highest prevalence [2]. An urgent surgical procedure should be carried out if ovarian torsion is detected in order to prevent permanent damage and preserve fertility.

Here, we present a case of a woman who presented with lower abdominal pain and was diagnosed with right adnexal torsion. While performing ovarian cystectomy with shortening of the right utero-ovarian ligament, the contralateral adnexa was incidentally found in the form of a pelvic mass sitting freely in the cul-de-sac, which could have been the result of a complete adnexal torsion that went undetected.

## Case presentation

A 25-year-old G0P0 woman presented to the emergency department with lower abdominal pain that started six days ago. It was described as a sharp pain that was intermittent and non-radiating. There was no associated nausea or vomiting. She also denied any headache, urinary complaints, cough, or chest pain. She was not sexually active. The patient was febrile with a temperature of 38.2^o^C and tachycardic with a pulse rate of 120 bpm; the remaining vital signs were within the normal range. On physical examination, there was no abdominal tenderness. Laboratory investigations revealed that her hemoglobin level was 6.8 g/dL and both activated partial thromboplastin time (APTT) and international normalized ratio (INR) was increased to 44.6 seconds and 1.45 seconds, respectively (Table 1). A bedside ultrasound was done, which showed a cyst in the right ovary measuring 5x5 cm, with no vascularity noted, suggestive of ovarian torsion while the left ovary could not be visualized.

**Table 1:** Laboratory investigations obtained preoperatively hCG: human chorionic gonadotropin, WBC: white blood cell, RBC: red blood cell, INR: international normalized ratio, APTT: activated partial thromboplastin time

Laboratory investigation	Laboratory value	Normal reference range
Beta hCG	<5 mIU/mL	Non-pregnant: < 5 mIU/mL, Pregnant: >5 mIU/mL
Complete blood count		
WBC count	9.1 k/uL	3.6-11 k/uL
RBC count	3.24 MIL/uL	3.80-4.80 MIL/uL
Hemoglobin, blood	6.8 g/dL	12-15 g/dL
Hematocrit	21.5%	36%-46%
Platelet count	338 k/uL	150-410 k/uL
Coagulation profile		
Prothrombin time	17.5 secs	11.5-14.5 secs
INR	1.45	0.80-1.20
APTT	44.6 secs	28.6-38.2 secs
Fibrinogen	429 mg/dL	190-430 mg/dL

**Figure 1:**
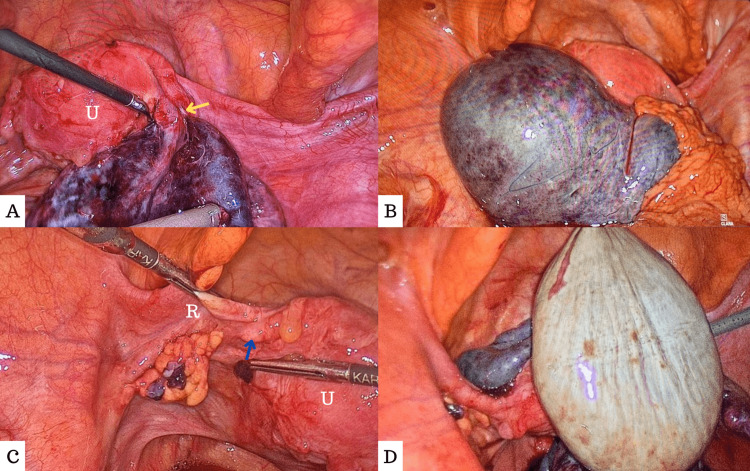
A. Right adnexa looking dark dusky bluish in color with the area of separation (yellow arrow); B. Right ovary, which is blackish in color and almost necrotic; C. Left adnexa showing a remnant proximal segment of the left fallopian tube (blue arrow) while the left grasper holds the round ligament (R); D. Left pelvic mass sitting freely in the cul-de-sac U: Uterus

A computed tomography scan of the abdomen and pelvis done at another facility showed a picture consistent with that of right-sided ovarian cyst torsion. Hence, the patient was taken for laparoscopic right ovarian cystectomy with shortening of the right utero-ovarian ligament. Intraoperatively, there was free fluid in the pelvis, and the right fallopian tube was found to be twisted, enlarged, edematous, and dark blue in color. At the area of torsion, the fallopian tube was divided and separated, and replaced with a fibrotic band (Figure 1A). The right ovary looked almost necrotic and blackish in color and contained two large cysts, 4-5 cm each, one hemorrhagic and the other containing clear-colored fluid (Figure 1B). After untwisting of the right adnexa, ovarian cystectomy was done while preserving the remaining ovarian mass as well as its attached divided fallopian tube. On the left side, neither the left ovary nor the left fallopian tube was visualized; instead, there was a remanent proximal segment of the left fallopian tube (Figure 1C). A free yellow-colored round pelvic mass was found sitting free in the cul-de-sac not adherent to the surrounding structures, which was thought to be the left adnexa that possibly underwent a complete torsion in the past, went unnoticed, and got amputated and separated from its pedicle (Figure 1D). The mass was then removed and sent for histopathology analysis and confirmation.

Intraoperatively, she was given three units of packed red blood cells and two units of fresh frozen plasma in view of her severe anemia. The histopathological analysis revealed that the left pelvic mass was indeed the left ovary that contained cystic infarcted and hemorrhagic tissue along with calcifications. The patient was discharged on postoperative day four in stable condition.

During her follow-up visit at three months postoperatively, she denied any hypoestrogenic symptoms such as hot flushes, and vaginal dryness. Her laboratory investigations during the visit showed a clinical picture of ovarian failure with elevated estradiol, follicle-stimulating hormone levels, and a decreased anti-Mullerian hormone level (Table 2).

**Table 2:** Laboratory investigations obtained three months postoperatively

Laboratory investigation	Laboratory value	Normal reference range
Anti-mullerian hormone	<0.01 ng/mL	1.20 - 9.05 ng/mL
Estradiol	29 pmol/L	Follicular phase: 114 - 332 pmol/L Mid-cycle phase: 222 - 1959 pmol/L Luteal phase: 222 - 854 pmol/L Post-menopause: 0 - 505 pmol/L
Follicle-stimulating hormone	161 mIU/mL	Follicular phase: 3.5 - 12.4 mIU/mL Ovulation phase: 4.7 - 21.5 mIU/mL Luteal phase: 1.7 - 7.7 mIU/mL Post-menopause: 25.8 - 135 mIU/mL

**Figure 2:**
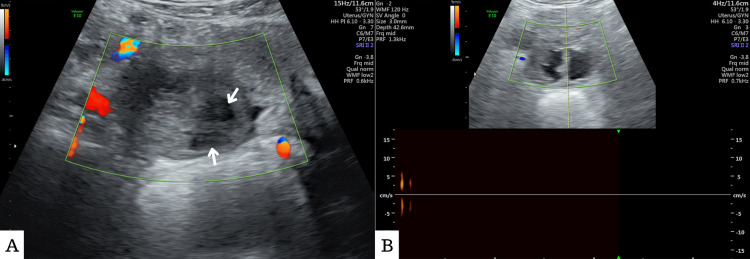
Ultrasound scan A. Two cystic spaces (white arrows) in the right ovarian tissue. B. No vascularity with color Doppler

An ultrasound scan was done, which showed enlarged ovarian tissue on the right side measuring 5.5 x 3.2 cm and a heterogenous appearance, with two irregular cystic spaces measuring 1.6 x 1.3 cm and 1.5 x 1.4 cm, respectively (Figure 2A). No vascularity could be detected by the application of the color Doppler (Figure 2B).

She was advised to start hormone replacement therapy to induce her periods as well as prevent osteoporosis and cardiovascular complications. However, the patient refused to do so. Surprisingly, during her one-year postoperative visit, the patient reported that she began having periods soon after the three months postoperative visit, though it was irregular and occurred every 90 days. Laboratory investigations were performed at her one-year postoperative visit, which demonstrated some improvement in her follicle-stimulating hormone (FSH) level and her estradiol level (Table 3). The patient was then advised for regular follow-up to monitor her progress.

**Table 3:** Laboratory investigations obtained one year postoperatively

Laboratory investigation	Laboratory value	Normal reference range
Anti-mullerian hormone	<0.01 ng/mL	1.20 - 9.05 ng/mL
Estradiol	202 pmol/L	Follicular phase: 114 - 332 pmol/L Mid-cycle phase: 222 - 1959 pmol/L Luteal phase: 222 - 854 pmol/L Post-menopause: 0 - 505 pmol/L
Follicle-stimulating hormone	55.3 mIU/mL	Follicular phase: 3.5 - 12.4 mIU/mL Ovulation phase: 4.7 - 21.5 mIU/mL Luteal phase: 1.7 - 7.7 mIU/mL Post-menopause: 25.8 - 135 mIU/mL

## Discussion

Adnexal torsion attributes to 2.7% of all gynecological emergencies [3]. There is some evidence of association, especially with benign ovarian masses larger than 5 cm; nonetheless, ovarian enlargement for any reason might raise the risk of torsion [4]. Torsion is specifically recognized to be a possibility with big, heavy cysts such as those observed in polycystic ovarian syndrome and after ovarian stimulation [4]. It is possible to misdiagnose adnexal torsion as other conditions that cause lower abdominal pain like renal colic and appendicitis [2]. Missing the diagnosis of adnexal torsion would lead to irreversible damage in the form of necrosis of the affected ovary, possible sepsis, and eventual death.

In an emergency, the most commonly employed imaging technique for assessing acute abdominal pain is ultrasonography. Large ovaries (> 4 cm) from early hemorrhage and edema are the most common findings [5]. The "string of pearls" sign, a concurrent mass inside the afflicted ovary, free pelvic fluid, and a twisted vascular pedicle are other typical ultrasonography signs [5]. Recent studies suggest that color Doppler sonography might be a helpful technique for improving the preoperative diagnosis of adnexal torsion. Although, it has been shown in several studies that the Doppler finding has a large false-negative rate [2]. The significant false-negative rate may be due to the varying degree of vascular impairment caused by the extent of tightness, number of twists, and length of torsion which can result in partial or total vascular disruption. As a result, the choice to do surgery should be based not just on the Doppler findings but also on clinical suspicion of adnexal torsion [2].

The safest method for treating adnexal torsion in women is by laparoscopy [2]. Laparoscopy has several advantages, including a decreased risk of wound-related issues, less postoperative discomfort and ileus, fewer hospital stays, decreased adhesion development, and a quicker return to regular activities. Ovarian function is found to be preserved in 88-100% of instances of adnexal torsion [6]. Intraoperatively, the viability of an ovary after torsion is often assessed grossly by visual inspection. An enlarged, dark ovary is likely to have lymphatic and vascular congestion. Ovaries with this appearance have often been assumed to be nonviable. However, multiple studies have discovered that after detorsion, such ovaries still maintain their function [7-9]. Recent studies have shown that conservative procedures like detorsion, either with or without cystectomy, favor retaining ovarian function [10]. Oophoropexy too has been shown to be effective in treating and preventing the recurrence of adnexal torsion, particularly in individuals with polycystic ovaries. Additionally, some studies have revealed that conservative detorsion of the adnexa did not affect antral follicle count in some patients [4,11].

Similarly, the patient mentioned in this case report presented six days after the onset of the lower abdominal pain. Intraoperatively, due to the delayed presentation, the initial impression was that the ovary was unsalvageable, as it looked necrotic as well. However, since the patient had only one remaining ovary, the decision of proceeding with a conservative approach was made. Postoperatively, the decision proved to be of good judgment, as the patient regained some ovarian function as the FSH and estradiol levels showed improvement and the patient began having periods, though irregularly.

The most commonly used markers of ovarian reserve that can help predict a woman’s ability to conceive in the future include FSH, estradiol, and anti-Mullerian hormone (AMH). Women under the age of 40 who have had significant ovarian tissue damage due to bilateral ovarian torsion are at risk of developing premature ovarian insufficiency (POI). It is diagnosed with an elevated FSH level (>40 IU/mL) on two separate occasions at least four weeks apart [12]. However, despite a clinical picture of POI, some women may regain ovarian function over time, such as the patient mentioned in this case report. There have also been some cases of spontaneous pregnancy even years after a diagnosis of POI [12].

The patient mentioned in this case report was nulliparous and was anxious to conceive in the future. Hence, such patients with bilateral adnexal torsion must be counseled, both preoperatively as well as postoperatively regarding the possibility of not regaining ovarian function and therefore losing their ability to conceive in the future. Such a situation may be traumatic to individuals who have not yet completed their families, and they must be appropriately referred to mental health services if needed.

## Conclusions

Acute lower abdominal pain in a female patient should warrant the need to consider adnexal torsion as a differential diagnosis. If there is a clinical suspicion of adnexal torsion, a decision on surgery should be taken as soon as feasible. Doppler flow analysis frequently produces false negative results. In addition to preserving ovarian function, conservative procedures like detorsion with cystectomy can also lower the recurrence rate. More studies have to be done to evaluate the long-term consequences on ovarian function and fertility following both oophoropexy and preservation of adnexa with delayed presentation of adnexal torsion.
